# Association of body mass index with mortality and postoperative survival in renal cell cancer patients, a meta-analysis

**DOI:** 10.18632/oncotarget.24210

**Published:** 2018-01-12

**Authors:** Jiao Zhang, Qiang Chen, Zhan-Ming Li, Xu-Dong Xu, Ai-Fang Song, Li-Shun Wang

**Affiliations:** ^1^ Institute of Fudan-Minhang Academic Health System, Minhang Hospital, Fudan University, ShanghaI 201199, China; ^2^ School of Public Health Taishan Medical University, Taian, Shandong 271000, China

**Keywords:** renal cell cancer, BMI, postoperative survival, mortality, meta-analysis

## Abstract

Obesity is one of the major risk factors of cancer. However, how body mass index (BMI) influences the prognosis of renal cell cancer (RCC) patient is unclear. In this work, we have performed a meta-analysis to elucidate the role of abnormal weight in RCC mortality and postoperative survival. Articles related to BMI and RCC mortality as well as postoperative survival has been identified by searching PUBMED and ENBASE. Totally, 19 articles have been selected for this meta-analysis, 5 articles for RCC mortality and 14 for postoperative survival. Compared to normal weight, the estimated relative risks of RCC mortality are 0.71 (95% CI: 0.34–1.49), 1.19 (95% CI: 1.05–1.35) and 1.71 (95% CI: 1.27–2.00) respectively for the underweight, overweight and obesity patients. The risk of RCC mortality increase 5% for each 1 kg/m^2^ increment of BMI. However, the estimated hazard ratios of cancer specific postoperative survival are 2.62 (95% CI: 1.67–4.11), 0.72 (95% CI: 0.63–0.83) and 0.66 (95% CI: 0.49–0.89) respectively for underweight, overweight and obesity RCC patients. The risk of hazard ratio decrease 5% for each 1 kg/m^2^ increment of BMI. In addition, the hazard ratios of postoperative overall survival show a similar tendency. These results indicate an opposite association of BMI with mortality and postoperative survival in renal cell cancer patients.

## INTRODUCTION

Cancer is one of the major causes of death in the world and an estimated 12.7 million new cancer cases occur annually, of which approximately 271,000 is new cases of kidney cancer [1]. Renal cell carcinoma (RCC), accounting for 2%-3% of all adult malignancies, is the most common kidney malignancy [2]. In many countries, RCC is increasingly diagnosed at an early stage, however, nearly 50% of RCC patients die within 5 years after diagnosis [3]. Obesity, hypertension and smoking are known risk factors of RCC [4–10]. Increased BMI was reported to be associated with high RCC incidence [11–13] both in men and women [14], however, extreme obesity didn’t predict poor cancer outcomes after surgery in RCC patients [15], which indicated that the association between BMI and RCC prognosis is controversial. Therefore, we performed a meta-analysis to explore the relationship between BMI and RCC mortality as well as postoperative survival.

## MATERIALS AND METHODS

### Literature retrieval

We conducted a literature search of PubMed and Embase database as well as hand-searching to 2017/07/30 for studies evaluating the effect of BMI on the risk of RCC mortality and postoperative survival. The studies were searched using the terms “BMI or body mass index or obesity or overweight” matched “mortality, prognostic or survival” and “renal cell cancer/carcinoma or kidney cancer/carcinoma”. And the publication language was restricted to English. We also searched the reference list of eligible articles. Only the most recent and informative one was included if several articles based on one data.

### Eligibility criteria

The criteria of studies included in this meta-analysis were set out as: (i) the exposure of interest should be BMI, weight, obesity or underweight; (ii) the outcome of interest should be mortality or postoperative survival; (iii) articles should report BMI categories and risk estimates with responding 95% CIs, or have sufficient information for us to calculate them.

### Data extraction

All the articles were analyzed independently by two investigators. Data extracted by one and checked by two. And discrepancies were discussed by all investigators until consensus was reached. The following information were extracted from each articles included: the first author’s name, publication year, country, mean age or median age and the range, follow-up year, study size, number of cases (Table 1), BMI categories and hazard ratio or relative ratio estimates including 95% CIs and surgical method (Table 2). We extracted RR or HR estimates from multivariate for analysis which were adjusted by most complete confounding factors for studies.

**Table 1:** Characteristics of the 5 included articles on BMI and mortality of RCC

Author, year country	Study type	Mean/ median age,	Follow up	Study size, number of cases	BMI (Kg/m^2^) cases	RR (95% CI)	Adjustment factors	NOS
Reeves et al. 2007 UK	Cohort	50–64	7	1222630	< 22.5 63 22.5–24.9 81 25.0–27.4 80 27.5–29.5 58 ≥ 30 100 per 10 units	1.01 (0.79–1.30) 1.00 (0.80–1.24) 1.14 (0.92–1.42) 1.30 (1.01–1.68) 1.71 (1.39–2.09) 1.65 (1.28–2.13	Age, socio economic status, smoking, alcohol, physical activity, region, years since menopause and use of hormone replacement therapy, geographical reproductive history,	7
Batty et al. 2005 Austrilia	Cohort	55.9	35	18403	18.5–24.9 36 25.0–29.9 20 ≥30 5	1.0 0.58 (0.32–1.04) 1.20 (0.41–3.52)	Age, physical activity, plus employment grade, smoking, marital status, disease at entry, weight loss in the last year, height, FEV1, blood pressure-lowering medication, triceps skin fold thickness, systolic blood pressure, plasma cholesterol, glucose intolerance and diabetes status.	8
Calle et al. 2003 USA	Cohort	57	16	900053 M:404576 W:495477	M 18.5–24.9 305 25.0–29.9 437 30.0–34.9 81 ≥ 35 14 W 18.5–24.9 243 25.0–29.9 153 30.0–34.9 55 35.0–39.9 12 ≥ 40 10	M 1.00 1.18 (1.02–1.37) 1.36 (1.06–1.74) 1.70 (0.99–2.92) W 1.00 1.33 (1.08–1.63) 1.66 (1.23–2.24) 1.70 (0.94–3.05) 4.75 (2.50–9.04)	Age, education, smoking status, physical activity, number of cigarettes, fat consumption, alcohol, marital status, aspirin use, vegetables consumption.	8
Parr et al 2010 Asia-Pacific	Cohort	48	4	424519	12.0–18.4 2 18.5–24.9 29 25.0–29.9 27 30.0–60.0 9 Per 5 units (> 18.5)	1.17 (0.28–4.97) 1.00 (0.70–1.43) 1.42 (0.96–2.12) 1.59 (0.78–3.24) 1.20 (0.86–1.66)	Age and smoking	7
Heath et al. 1997 USA	Cohort	56–57	7	998904	M < 20.7 4 20.7–24.6 62 24.7–27.7 81 27.8–31.0 48 ≥31.1 17 W < 19.1 2 19.1–21.9 18 22.0–27.2 57 27.3–32.2 33 ≥ 32.3 13	M 0.6 (0.2–1.5) 1.0 1.1 (0.8–1.6) 1.6 (1.1–2.3) 1.6 (0.9–2.7) W 0.6 (0.1–2.5) 1.0 1.5 (0.9–2.6) 2.5 (1.4–4.4) 3.15 (1.5–6.4)	Adjusted age	7

**Table 2:** Characteristics of the 14 articles on BMI and postoperative survival of RCC

Author year country	Mean/ median age, range	Follow up year	Study size	*n*	BMI (kg/m2)	HR (95%CI)	surgical method	Adjustment factors	NOS
Sung et al 2012 Korea	median 54 (34–67) 54 (45–63) 56 (47–63)	4.57	1487	42 833 612	< 18.5 18.5–25 ≥ 25	CSS 2.17 (1.16–4.08) 1 0.66 (0.45–0.96)	radical partial	age, gender, anemia ASA score, cell type, tumor grade, T, N and M stage,	7
Teng et al 2014 China	mean 53.4 (41–65.8)	5	378	11 349	< 18.5 ≥ 18.5	CSS 5.812 (1.124–30.059) NA	radical partial	tumor necrosis, sarcomatoid change, high Ki-67 expression level, advanced Fuhrman grade, and T stage	8
Lee et al 2010 Korea	mean 54.9 (12–90)	2.3	2981	Na	< 30 ≥ 30	CSS NA 0.599 (0.146–2.456)	radical partial	age, sex, T stage, and Fuhrman’s grade	6
Haferkamp et al 2008 Germany	median 61.6 (14.6–89.0)	5.3	780	10 245 361 141	< 18.5 18.5–25 25–30 ≥ 30	CSS 4.27 (1.47–12.4) 1 1.00 (0.75–1.3 1.11 (0.74–1.65)	radical	age, gender, Karnofsky PS, tumour stage Fuhrman grade, histological type and BMI	7
Schrader et al 2009 Germany	mean < 25 64.9 (53.7–76.1) ≥ 25 62.6 (52.2–73)	5.48	771	4 239 356 172	< 18.5 18.5–25 25–30 ≥ 30	CSS NA 1 0.65 (0.50–0.86) 0.60 (0.42–0.84)	radical nephron–sparing laparoscopically	tumor grade, stage, lymphatic metastasis, pulmonary/visceral metastasis, histological subtype, age, sex, and tumor–related symptoms at presentation	7
Kamat et al 2004 US	mean 58.3 (19–85)	9.75	400	125 275	< 25 ≥ 25	CSS 1 0.46 (0.24–0.85)	Not available	Age, stage and grade	7
Jeon et al 2010 Korea	54.7 (20–83)	6.41	1017	363 526 128	< 23 23–27.5 ≥ 27.5	CSS 1 0.67 (0.46–0.98) 0.42 (0.19–0.89) OS 1 0.71 (0.51–0.99) 0.41 (0.21–0.80)	radical nephron sparing	age, BMI, pathological T stage, regional lymph node involvement, distant metastases, tumor size and sarcomatoid change	7
Choi et al 2013 Korea	54	3.67	1543	41 448 385 669	< 18.5 18.5–23 23–25 ≥ 25	CSS 2.13 (0.84–5.36) 1 1.00 (0.63–1.60) 0.47 (0.29–0.77) OS 2.05 (0.85–4.91) 1 1.02 (0.68–1.53) 0.45 (0.29–0.68	radical partial	Age, sex, WL, stage, size, NT, HS, grade Symptom, ESR	7
Komura et al 2011 Japan	62.4 (21–86)	4.17	170	83 87	< 22 ≥ 22	CSS 1 0.091 (0.009–0.904)	radical partial	Mode of presentation, ECOG PS, C–reactive protein, HS, grade, microvascular	6
Cho et al 2009 Korea	56	4.33	299		< 23 23–25 ≥ 25	CSS 1 0.510 (0.195–1.329) 0.200 (0.045–0.884)	radical nephron– sparing	Capscular invasion, stage	7
Donat et al 2006 US	61 (52–70)	9.17	1159	1137 278 472 387	< 25 25–30 ≥ 30	OS 1 0.69 (0.48, 1.00) 0.90 (0.62, 1.30)	radical partial	age type of operation, systemic symptoms at presentation	7
Steffens et al 2013 European	62.3 (20–90)	4.72	2030	700 885 445	<25 25–30 ≥ 30	CSS 1 0.71 (0.57–0.87) 0.94 (0.73–1.21)	radical partial	Age and sex, tumor stage, differentiation grade, histopathological subtape, lymphogenous/visceral metastasis	6
Ha et al 2011 Korea	TLRN: 56.5 (44.3–68.7) RLRN: 54.8 (43.0–55.6)	2.59	580		< 23 23–24.9 ≥ 25	OS 1.000 0.342 (0.093–1.263) 0.359 (0.113–1.139)	TLRN RLRN	age, sex, NT, ECOG PS, grade, stage	7
Lee et al 2015 Korea	55.9 (43.5–68.3)	3.17	2769	853 1916	18.5–23 ≥ 23	CSS 1 0.611 (0.441–0.847)	Not available	Age, gender, symptoms at presentation, Tumor size, T stage, Fuhrman’s grade, Histologic subtype.	7

### Assessment of methodological quality

We assessed the methodological quality of the articles included rely on the Newcastle-Ottawa Scale Star (NOS) system [16]. It assesses study quality by 3 classifications including selection, comparability and outcome with a total score of 9 stars. Of these 9 stars, 4 represents for the appropriate selection of exposure and nonexposure cohort participants, 2 represents for the comparability of cohort, and the last 3 describes the assessment of outcome and follow-up. Articles were considered as high quality study if its’ quality score more than or equal to 5 stars.

### Statistical methods

For different BMI levels, we conducted separate meta-analysis [14]. For each study, the normal weight represented the referent category, underweight represented the lowest category, obesity represented the highest category which provided that two or more categories above the reference category, whiles the overweight represented between obesity and normal weight categories. And we compared each categories with normal weight. The summary estimates of RR or HR and 95% CIs were calculated by using a random-effects model to evaluated the inter-study heterogeneity [17].

For dose-response analysis, a two-staged random-effects dose-response meta-analysis [18] was performed to compute the trend from the correlated logHR estimates across levels of BMI, taking into account the between-study heterogeneity. Firstly, a restricted cubic spline model with three knots at percentiles 10, 50 and 90% of the distribution was estimated using generalized least-square regression taking into account the correlation within each set of published RRs/HRs. Then, the GLST command with the generalized least-squares regression was used to carry out the dose-response meta-analysis. The estimated RR/HR and their 95% CI were represented by solid line and the long dash line. Short dash line represents the liner relationship (per 1 kg/m^2^ increment). And a *p* value for nonlinearity was calculated by testing the null hypothesis that the coefficient of the second spline was equal to zero [19]. And the evidence of publication bias was estimated by visual inspection of funnel plots using Egger’s regression test [20].

All the statistical analysis was performed by using Stata SE 12 for Windows (Stata Corporation, College Station, TX). And all *p* values were 2-sided. *p* < 0.05 was regarded as statistically significant.

## RESULTS

### Characteristics of the studies

**Figure 1:**
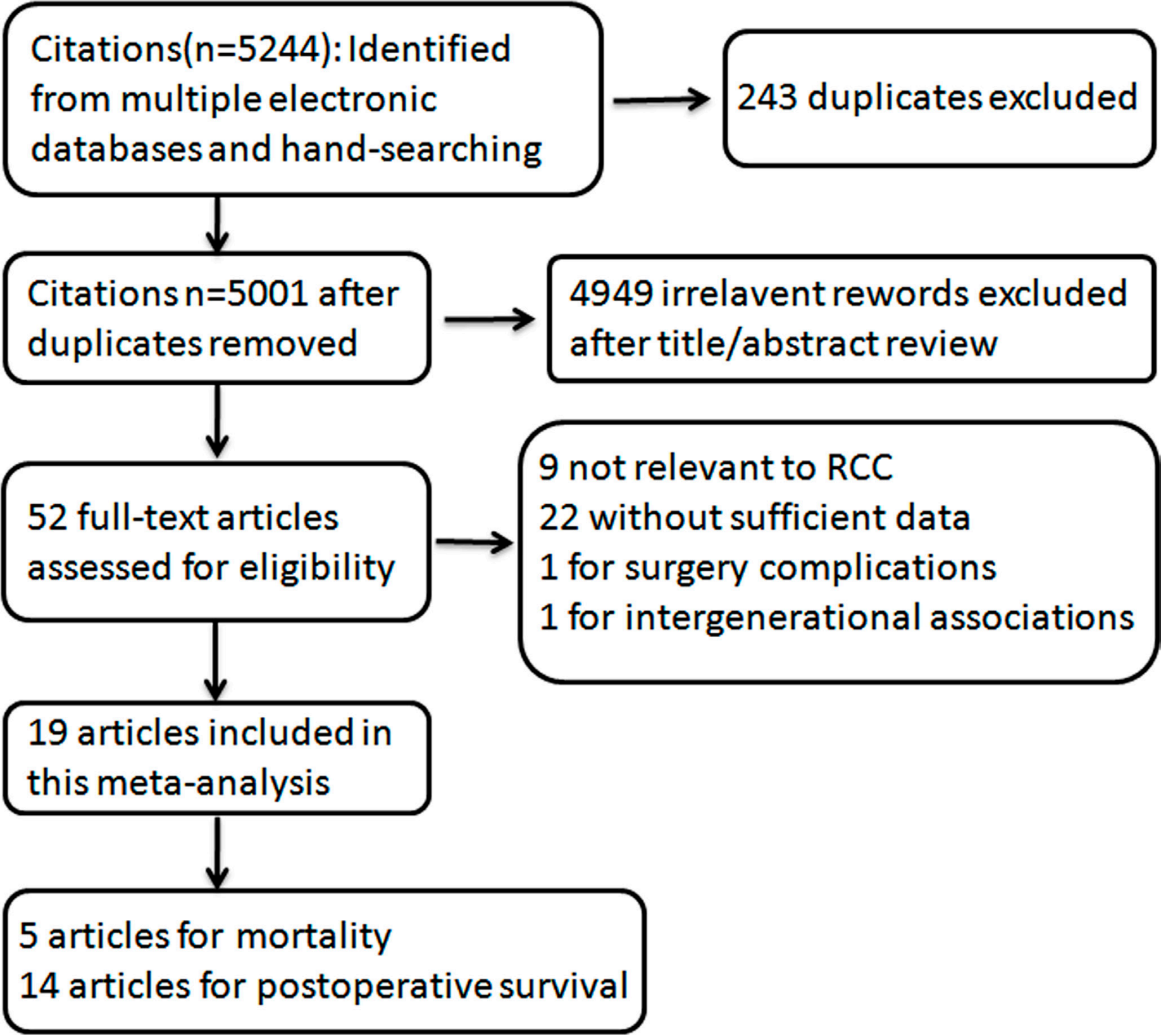
Flowchart of selection of studies for inclusion in this meta–analysis

5244 citations were identified from PUBMED and EMBASE, of which 243 duplicative ones were excluded after reviewed. Figure 1 showed the process of our study selection. As for the remaining 5001 citations, 4949 were excluded by scanning either the titles or abstracts. For the 52 remaining potentially related articles, full-text was reviewed in detail, of which 9 articles not relevant to RCC, 22 without sufficient data reported, 1 for surgery complications and 1 for intergenerational associations were excluded. Totally 19 articles were included for this meta-analysis, of which, 5 for RCC mortality [21–25], and 14 for RCC postoperative survival [26–39]. The follow-up intervals were from 4 to 35 years for RCC mortality, and 2.3 to 9.75 years for RCC postoperative survival.

The main characteristics of the studies are shown in the Table 1 and Table 2. Newcastle-Ottawa Scale (NOS) was used to assess the quality score of these articles and all these included articles were above 6 stars (Tables 1 and 2).

### BMI and mortality in RCC

**Figure 2:**
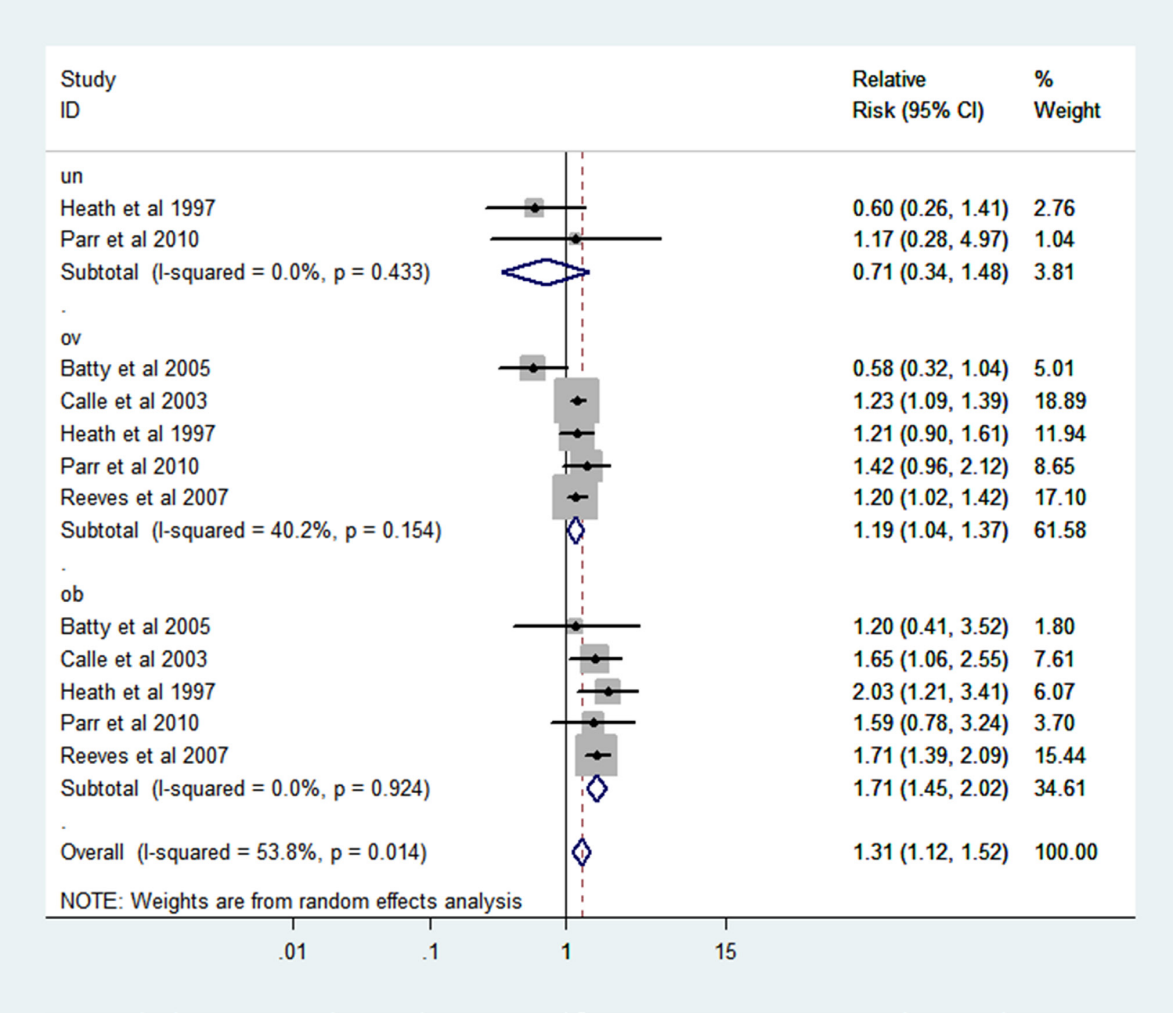
Forest plot of RR of abnormal VS normal weight for BMI with RCC mortality

The association of BMI and RCC mortality was shown in Figure 2. With the comparison to normal weight, the estimated relative risk (RR) of RCC mortality for the underweight was 0.71 (95% CI: 0.34–1.48), while for the overweight and obesity, the RRs were 1.19 (95% CI: 1.04–1.37) and 1.71 (95% CI: 1.45–2.02) based on random-effect models. There was no heterogeneity in underweight and obesity groups and the heterogeneity was slightly high in overweight group (I^2^ = 40.2%, *P* = 0.154).

**Figure 3:**
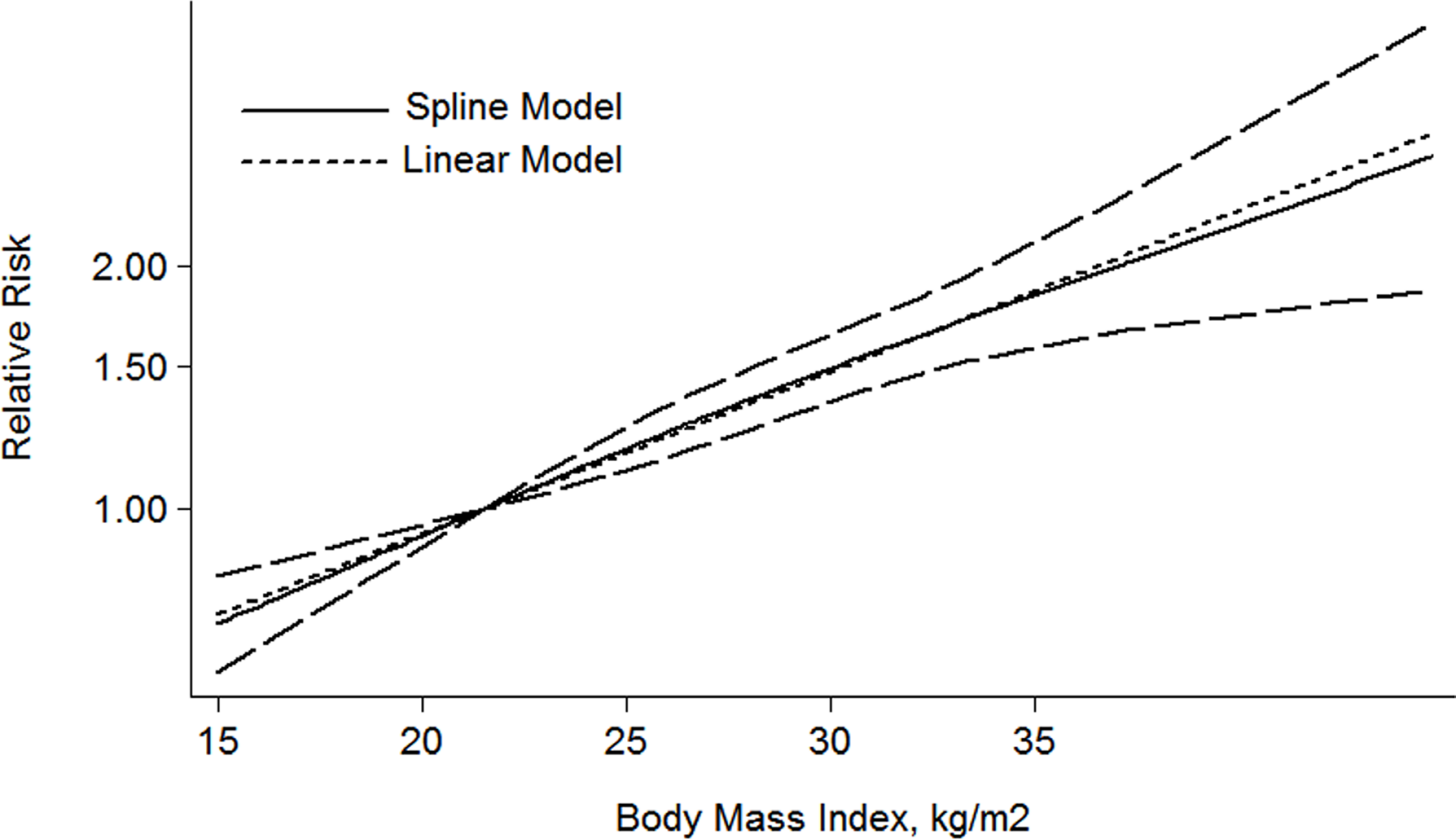
The dose-response analysis between BMI and RCC mortality in studies with restricted cubic spline in a multivariate random-effects dose-response model

In addition, a dose-response meta-analysis including 5 articles [21–25] was performed, a liner relationship was shown in the Figure 3. It was revealed that per 1 kg/m^2^ increment in BMI was associated with a 5% higher risk of mortality in RCC patients (RR: 1.05 (1.03–1.07) *P* = 0.000).

### BMI and postoperative survival in RCC

**Figure 4:**
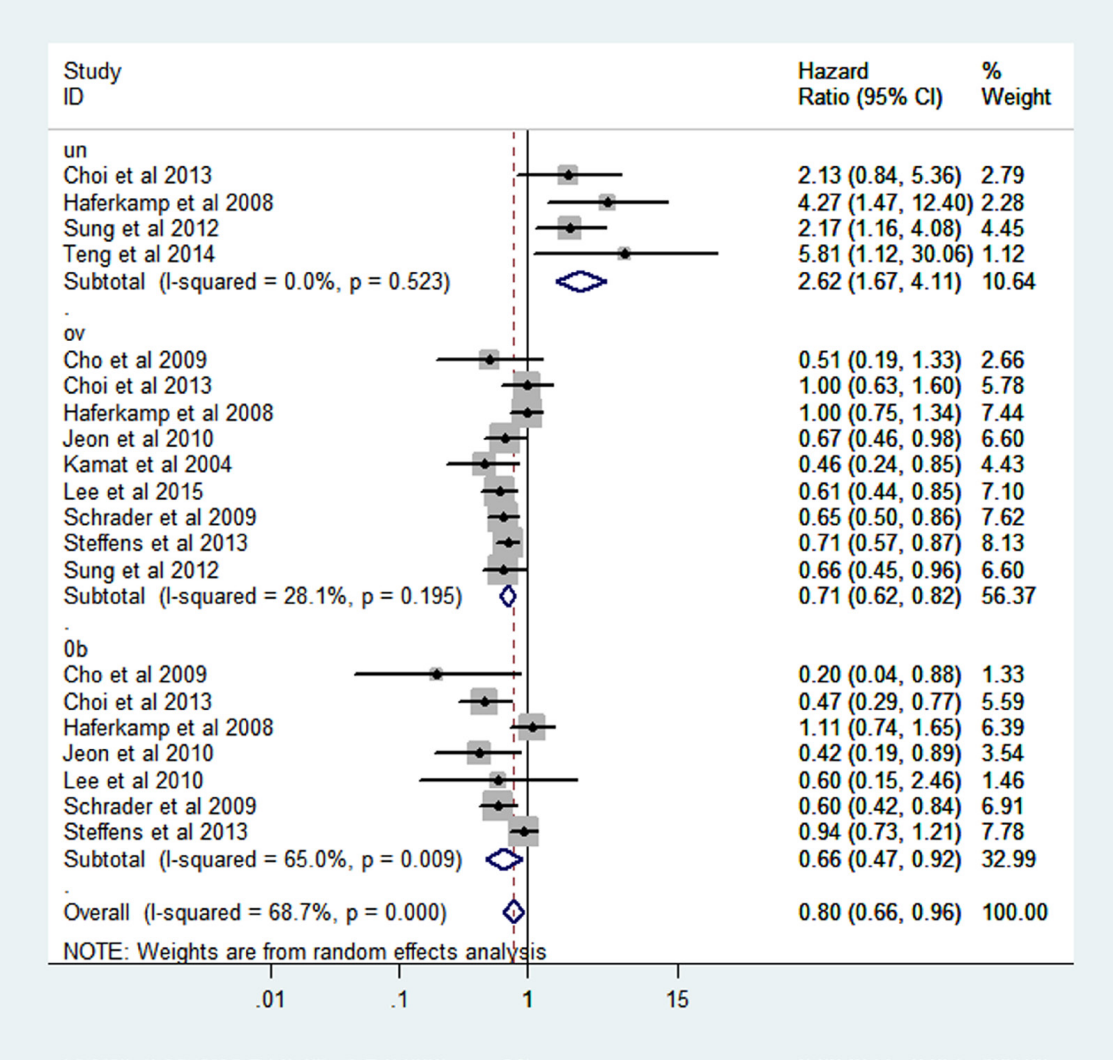
Forest plot of HR of abnormal weight VS normal weight for BMI with RCC CSS

The combined hazard ratio (HR) of CSS were 2.62 (95% CI: 1.67–4.11), 0.71 (95% CI: 0.62–0.82), 0.66 (95% CI: 0.49–0.89) respectively for the category of underweight, overweight and obesity based on random-effect models (Figure 4). Heterogeneity was found in obesity group (I^2^ = 65.0% *P* = 0.009).

**Figure 5:**
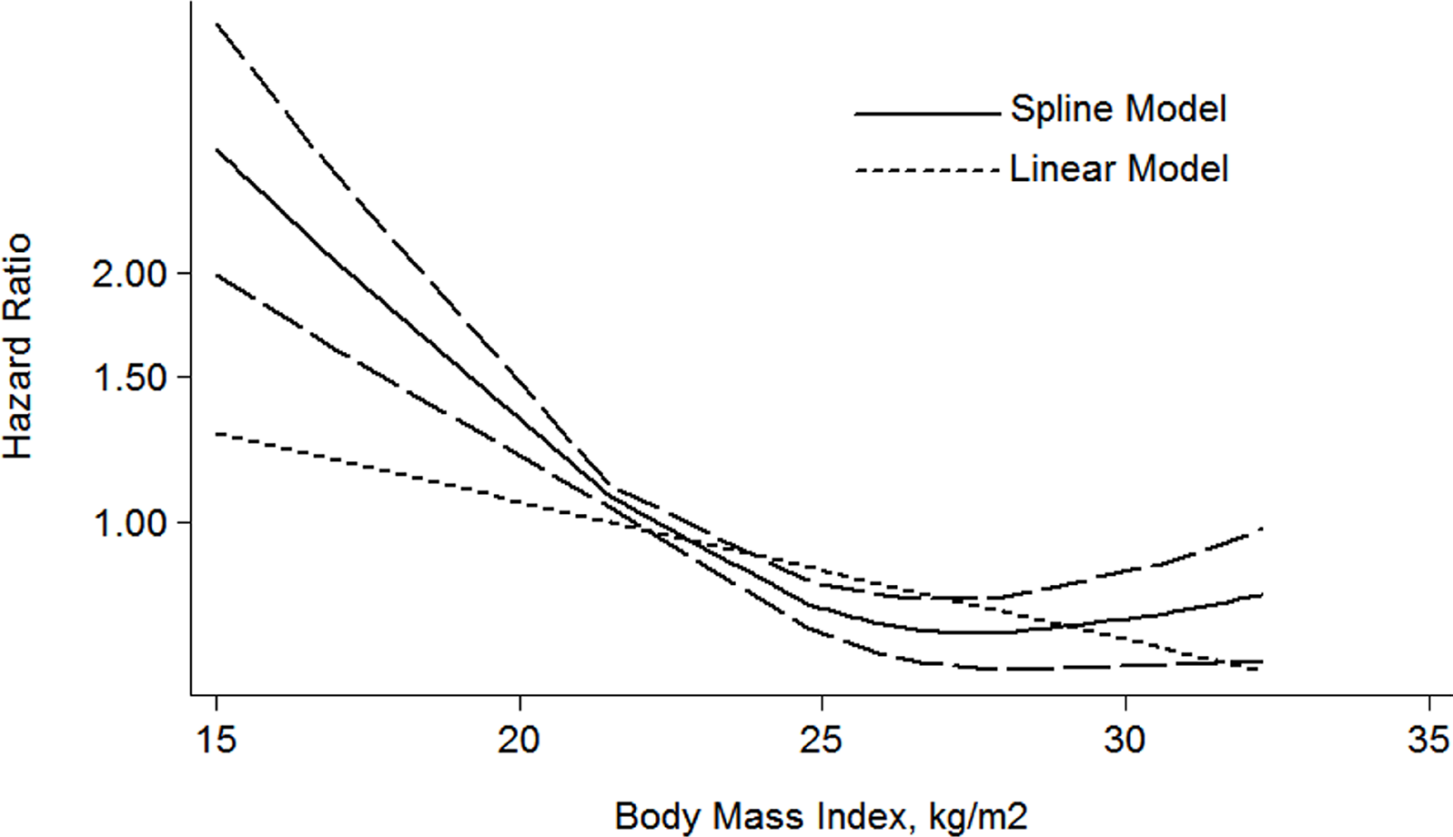
The dose-response analysis between BMI and RCC CSS in studies with restricted cubic spline in a multivariate random-effects dose-response model

For the dose-response meta-analysis, 6 articles were included [28, 30, 31, 34–36]. As shown in the Figure 5, the BMI-CSS relationship showed a L-shaped curve with a nadir at around BMI value of 26 kg/m^2^. The risk of HR decrease 5% for each 1 kg/m^2^ increment of BMI (HR: 0.95 (0.92–0.98) *P* = 0.001).

**Figure 6:**
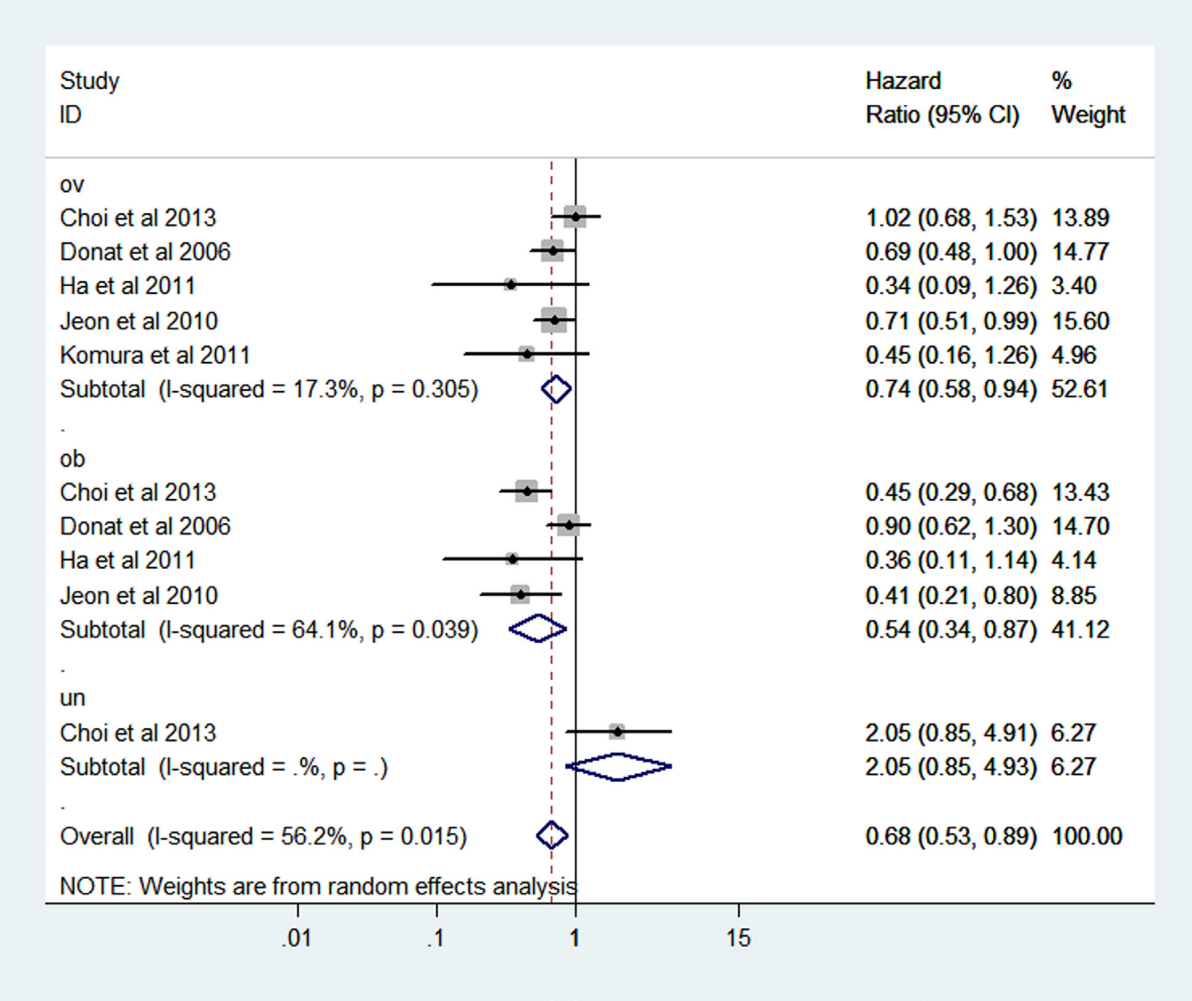
Forest plot of HR of abnormal weight VS normal weight for BMI with RCC OS

**Figure 7:**
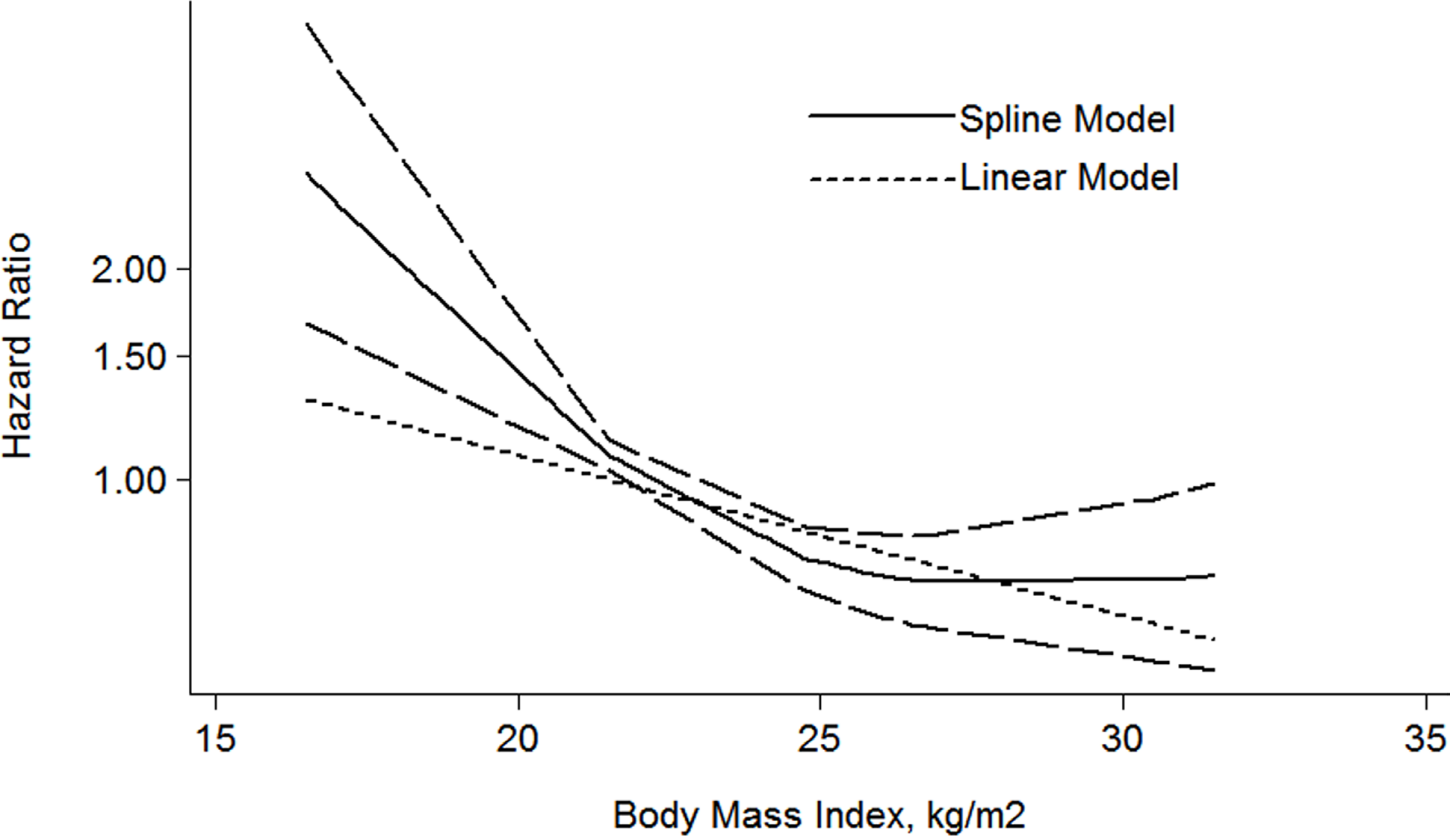
The dose-response analysis between BMI and RCC OS in studies with restricted cubic spline in a multivariate random-effects dose-response model

In addition, overweight and obesity patients had significantly longer overall survival (OS) time than normal weight patients (HR: overweight: 0.76 (95% CI: 0.61–0.95); obesity: 0.57 (95% CI: 0.34–0.97)) (Figure 6). For the BMI-OS dose-response meta-analysis, 4 articles were included [27, 31, 33, 35], which showed a similar tendency with the CSS curve (HR: 0.93 (0.86–0.99) *P* = 0.021) (Figure 7). Before the BMI of around 26 kg/m^2^, the HR of survival decreased with the increase of body weight, and then tended to a stable value.

### Meta-regression analysis

We conducted the meta-regression analysis to investigate whether the association between BMI and RCC postoperative survival is modified by study location, publication year, follow up and sample size. We found that the study location can explain 62.8% and 100% heterogeneity for CSS and OS in the obesity category. For the Asia, the combined HR were 0.44 (95% CI: 0.30–0.64; I^2^ = 0.0%) and 0.43 (95% CI: 0.31–0.61; I^2^ = 0.0%) for CSS and OS in the obesity category.

### Sensitivity analysis

For study influence analysis, one study was removed and the rest was analyzed. The pooled RR for mortality ranged from 0.60 to 1.17 for underweight, from 1.14 to 1.23 for overweight and from 1.68 to 1.73 for obesity respectively. And for postoperative CSS, the pooled HRs ranged from 2.36 to 3.20 for underweight, from 0.68 to 0.73 for overweight and from 0.58 to 0.71 for obesity respectively. The HRs for overweight and obesity ranged from 0.70 to 0.76 and 0.43 to 0.58 for postoperative OS. All of the results showed that the pooled estimates were stable and not influenced by a single study.

### Publication bias

Publication bias was assessed by using Egger regression test and Begg funnel plot. And there were no efficient evidence indicating publication bias on the relationship of BMI and RCC mortality and postoperative survival.

## DISCUSSION

In this meta-analysis, we observed a statistically significant increased risk of RCC mortality in overweight and obese individuals as compared with their normal-weight peers. However, decreased risk of postoperative survival was observed in overweight and obesity RCC patients.

RCC mortality is the measure of deaths (in general, or due to a specific cause) in RCC population. The mechanism by which obesity may increase RCC mortality is not well studied [40], although mechanisms linking obesity with RCC incidence have been studied long time. Increasing prevalence of obesity is thought to contribute to the increasing incidence of RCC via several hormonal mechanisms including free estrogen [41], insulin and IGF-1 [42, 43], as well as physical damage mechanisms by lipid peroxidation, higher glomerular filtration rate and renal plasma flow [44, 45]. The RCC progression might share these mechanisms and develop fast in obesity RCC patients. In addition, increased BMI also increase the risk of other chronic disease including cardiovascular and stroke and thus increase the death risk.

On the other hand, it was reported that people without surgery-treatment have a significantly shorter survival time than surgical patients [31, 46, 47] indicating that surgery is a great factor for RCC postoperative survival. Obesity contributes to poor postoperative complications especially wound infection [48–53]. Yet, overweight and obesity might provide more sufficient nutritional reserve and metabolic state to overcome the stress of surgery. On the other hand, underweight are bad in energy use and metabolic excess, which fail to deal with the extreme stress of major surgery as well as remained tumor cells. And a molecular mechanism underlying this phenomenon may be that higher BMI associating with lower serum total adiponectin and thus may inhibit the remaining disseminated RCC cells [54].

A clinical-based cohort and meta-analysis by Choi et al [35] revealed that the postoperative survival significantly increased in highest BMI RCC patients than lowest BMI RCC patients. To clearly reveal the relationship between each BMI category and RCC postoperative survival, the postoperative survival of abnormal weight (underweight, overweight and obesity) were compared to normal weight in this study, in addition, their dose-response relationship were analyzed and the risk of hazard ratio was found to decrease 5% for each 1 kg/m^2^ increment of BMI.

Previously, a meta-analysis by Bagheri et al [55], of which 6 articles included, 5 for CSS, 3 for OS, stated that the CSS increased in relation to BMI, while for the OS, it decreased for each increase in BMI over 25kg/m2. Of our meta-analysis, 14 articles have been included for CSS and OS, and the results indicate that both overweight and obesity were beneficial to postoperative survival. Our conclusion is not identical to this meta-analysis. The numbers of included articles for the meta-analysis may contribute to this difference. In addition, that meta-analysis didn’t study the relation between BMI and RCC mortality. Our meta-analysis have studied the relationship between BMI and RCC mortality and found that BMI was a risk factor for RCC mortality.

On the other hand, BMI and the incidence of RCC had been analyzed by wang et al [14], whose results indicated that increase of body weight may increase the incidence of RCC. The increased incidence of RCC in overweight and obesity populations might contribute to the corresponding increased mortality of RCC.

There are some limitations in our meta-analysis. Firstly, abnormal weight was associated with unhealthy diet habit, but the studies included were almost not adjusted for it. Secondly, obesity tends to be accompanied with diabetes, which is also associated with RCC [56], but there is no sufficient information to analyze it. Thirdly, lacking of higher obese, such as obese class III, information limit us to evaluate the status of morbidly obese survival. Last, the association between BMI and RCC histological information were lacked which limit us to histological subgroup analysis.

In conclusion, our meta-analysis indicated that obesity may be associated with high risk of mortality in whole RCC patient but a better survival in surgery-treated RCC patients. Individualized weight control might be necessary for RCC patients.
